# Psychometric Properties of the Children's Auditory Perception Test: Reliability and Validity Analysis

**DOI:** 10.1002/brb3.70301

**Published:** 2025-03-20

**Authors:** Ozlem Icoz, Selen Yilmaz Isikhan, Esra Yucel

**Affiliations:** ^1^ Department of Audiology Faculty of Health Sciences Hacettepe University Ankara Turkey; ^2^ Department of Biostatistics Faculty of Medicine Hacettepe University Ankara Turkey; ^3^ Vocational Higher School of Social Sciences Hacettepe University Ankara Turkey

**Keywords:** auditory perception, auditory rehabilitation, cochlear implant, reliability, validity

## Abstract

**Purpose:**

This study aimed to revise and investigate the validity and reliability of the Children's Auditory Perception Test (CIAT), which was developed to evaluate auditory perception skills.

**Methods:**

The study included 100 cochlear implant (CI) users between the ages of 2 and 15, and 80 individuals with normal hearing. In the first session, participants underwent the Turkish Early Language Development Test (TELD‐3) and audiometric assessments. The second session involved administering age‐appropriate subtests from the CIAT battery. Subtest reliability was evaluated using internal consistency and test–retest methods. We measured the construct validity by examining the relationships between subcategories. Also, we evaluated known‐group validity and predictive validity.

**Results:**

The reliability analysis of the CIAT indicated high internal consistency, with a Cronbach's alpha coefficient of 0.913 for all 17 tests. Subcategories demonstrated reliability ranging from acceptable to excellent (*α* = 0.741–0.973). Significant differences were observed in auditory perception scores between children with CI and those with normal hearing (*p* < 0.005), demonstrating the known‐group validity of the test across different age groups. Multiple linear regression analysis revealed that factors such as age group, gender, special education duration, receptive and expressive language ages, CI duration, and usage status accounted for 78% of the variability in auditory perception scores (*R* ^2^ = 0.78), thus testing the predictive reliability of the model.

**Conclusion:**

A valid and reliable test battery that evaluates auditory perception skills at different difficulty levels across a wide age range (2–15 years) has been introduced to the literature. However, a notable limitation is that this battery does not include auditory processing assessments, such as speech‐in‐competition (noise/babble) tests, which could enhance the comprehensiveness of the evaluation.

## Graphical abstract

The CIAT distinguishes between children with cochlear implants and normal‐hearing peers in auditory perception. Scores are influenced by age, CI usage duration/status, and language development, with CI duration being most significant. CIAT is a validated, reliable tool assessing auditory skills like detection, recognition, and comprehension for ages 2–15.

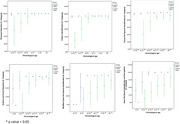

## 1 Introduction

With the widespread implementation of newborn hearing screening, hearing loss is diagnosed at an early stage. Early intervention, coupled with the use of suitable amplification systems, has led to significant improvements in the language and auditory perception skills of children with hearing impairment. It is crucial for rehabilitative audiologists to regularly assess the progress of auditory perception skills to gauge the effectiveness of the therapy program. Auditory perception tests can be utilized alongside other measurements to assess, guide habilitation, and reveal rehabilitation needs of children with hearing loss by identifying developed, emerging, and lost auditory and speech perception areas (Kirk, Pisoni, and Osberger 1995).

Various assessments have been developed to evaluate auditory perception skills and overall auditory skills in children with hearing loss. Auditory perception tests commonly used in the literature include the Northwestern University‐Children's Perception of Speech (NU‐CHIPS) (Elliott and Katz 1980), Early Speech Perception (ESP) (Moog et al. 1990), Pediatric Speech Intelligibility (PSI) (Jerger et al. 1980), Word Intelligibility by Picture Identification (WIPI) (Ross, Lerman, and Cienkowski 2004), and Evaluation of Auditory Responses to Speech (EARS) (Allum‐Mecklenburg 1996). However, except for the EARS, these tests have not been adapted into Turkish. The EARS has been adapted into more than 20 languages, including Turkish. However, it is crucial to develop language‐specific test batteries and conduct validity studies to ensure accurate assessment in different linguistic contexts.

To the best of the author's knowledge, several tools are available in Turkish for assessing auditory perception in children, including questionnaires like the Meaningful Auditory Integration Scale (MAIS) (Robbins, Renshaw, and Berry 1991), the Infant‐Toddler Meaningful Auditory Integration Scale (IT‐MAIS) (Zimmerman‐Phillips, Robbins, and Osberger 2000), the Auditory Behavior in Everyday Life Questionnaire (ABEL) (Özses et al., 2022), the Parents’ Evaluation of the Aural/Oral Performance of Children (PEACH) (Ching and Hill 2007), and the Children's Auditory Performance Scale (CHAPS) (Baydan et al. 2020), as well as speech‐in‐noise tests such as HINT‐C (Kartal Özcan et al. 2023). However, the Children's Auditory Perception Test (CIAT) remains the only test battery specifically designed to assess fundamental auditory abilities in a hierarchical manner, focusing on detection, identification, and recognition skills (Yucel and Sennaroglu 2011).

CIAT was developed in Turkish to evaluate the auditory perception skills of children aged 2–15. CIAT has been widely used in many studies (Yücel et al. 2015; Ozkan et al. 2021, 2022; Yucel, Sennaroglu, and Belgin 2009; Aslan et al. 2020). However, several disadvantages have been identified in clinical use, highlighting the need for revisions and the addition of supplementary tests to the test battery. It has been observed that a number of the pictures in the test are outdated, and several test items have been reported to be unrecognizable by children today. Some examples of outdated items include pictures of a stamp and a gas cylinder. Also, the validity and reliability studies were conducted on a small sample size, and there is a lack of normative data for normally hearing children. The Open‐Set Sentence Identification Test, consisting of 2–3‐word sentences, is too easy for older children, and the recognition test only includes single‐command phrases, which do not adequately evaluate memory skills. These limitations underscore the need to revise the existing test and update the stimuli to better reflect cultural changes, as well as to incorporate additional tests to enhance the overall assessment.

While updating this test battery that evaluates auditory perception abilities, our aim is to use a language appropriate to the native language and to choose words and sentence structures that children learn to use in daily language. This allows for more accurate and effective assessment of children. In addition, using the native language can help obtain more reliable results by better reflecting the language patterns that children encounter in daily life.

The aim of our study is to revise the CIAT and investigate its validity and reliability. Through this, we aim to provide a test battery for evaluating auditory perception skills that can be used in clinical and research settings.

## 2 Materials and Methods

This study was approved by the ethical committee of Hacettepe University (GO 17/991 2018/03‐20). Before beginning the study, all parents and children were given verbal and written information about the purpose of the study, and their written informed consent was obtained.

### 2.1 Participants

Overall, 180 participants aged 2–15 were included in the study, comprising 100 cochlear implant (CI) users and 80 normally hearing children. When determining the sample size, the rules of thumb were applied, which suggest that the ratio of participants (*N*) to measured variables (*p*) should be considered. According to this guideline, the sample size must be greater than the number of variables (*N* > *p*) (Dimitrov 2014). A widely accepted standard recommends including 5–10 times the number of variables in most studies (Wang and Wang 2019; Nunnally, Bernstein, and Berge 1967). In this study, considering the subtest with the highest number of items, the ratio is calculated as 7 participants per item, resulting in a total of 175 participants (25 items × 7 participants per item), exceeding the recommended minimum threshold.

Participants with CIs had undergone surgery at the Department of ENT at Hacettepe University Hospital and were regularly followed up in the Department of Audiology. Children who visited the audiology department for a hearing test and were found to have normal hearing were included in the control group.

Inclusion criteria for the study group were: (a) prelingual children with bilateral severe to profound sensorineural hearing loss who had unilateral or bilateral CIs, (b) no neurological or physiological disorders in their medical records, (c) no history of meningitis, auditory neuropathy spectrum disorder, or inner ear malformation, (d) received speech, language, and auditory training postcochlear implantation, (e) activated speech processor for at least 1 month, (f) exposed only to the native language (Turkish), and (g) hearing thresholds with CIs within the speech range.

Inclusion criteria for the control group were: (a) bilateral normal hearing, (b) no neurological or physiological problems in their medical records, and (c) exposure only to the native language (Turkish).

There was no significant difference between the gender (*p* = 0.283) and chronological age (*p* = 0.756) between the study and control groups. Demographic characteristics of the study and control groups are presented in Table 1.

**TABLE 1:** Demographic characteristics of the study and control groups.

		Study group Number (%) or mean (SD)	Control group Number (%) or mean (SD)
Gender	Female	52 (52)	48 (60)
	Male	48 (48)	32 (40)
Age group (month)	24–35	10 (10)	5 (6.3)
	36–47	10 (10)	10 (12.5)
	48–59	13 (13)	14 (17.5)
	60–71	15 (15)	11 (13.8)
	72–83	11 (11)	12 (15)
	84+	41 (41)	28 (35)
The average age		81.90 ± 38.57	77.43 ± 33.87
Receptive language		57.59 ± 31.4	73.78 ± 24.8
Expressive language		56.55 ± 32.5	75.81 ± 23.1
Duration of CI usage (month)	1–12	21 (21%)	
	12–24	8 (8%)	
	24–36	16 (16%)	
	36+	25 (25%)	
CI types	Unilateral	35 (35%)	
	Bimodal	17 (17%)	
	Bilateral	48 (48%)	
Age at CI (month)	12–18	21 (21%)	
	18–24	8 (8%)	
	24–36	16 (16%)	
	36+	25 (25%)	
CI brand	Nucleus	51 (52%)	
	Medel	38 (38%)	
	Advanced bionics	10 (10%)	
Age at diagnosis (month)		5.38 ± 7.8	
Age at hearing aid fitting (month)		9.50 ± 7.8	
Average age at CI (month)		32.44 ± 21.69	
Duration of special education attendance (year)		4.11 ± 3.1	

### 2.2 Study Design

This study was conducted in two phases:

In the first phase, the CIAT battery developed by Yücel et al. 2011 was revised to address current cultural needs and lifestyle changes in its content and imagery. During the preparation phase, various test batteries designed to evaluate auditory perception skills were reviewed to determine the content for the new test battery. The categories to be evaluated were identified and ranked according to difficulty. For each category, the necessary syllables, words, sentences, picture cards, and toys were selected, considering the words and toys familiar to children in their daily lives. In addition, feedback was gathered from three experts in rehabilitative audiology, and a pilot study was conducted with 20 native Turkish‐speaking children aged 3–5 with normal hearing to review the materials for acceptability and suitability as an assessment tool for the Turkish population.

In the second phase, the developed test battery was administered to CI users and normally hearing participants, starting with the first category and progressing through the levels according to their developmental stages.

The test battery, consisting of a total of 17 different tests across 6 categories ranging from easy to difficult, was designed to evaluate auditory perception skills in a hierarchical manner.

### 2.3 Material

The tests conducted as part of this study were carried out in two sessions. In the first session, a pure tone audiometry test was administered to the control group to exclude possible hearing losses and to determine if the hearing thresholds of the study group were within the speech range. In addition, the Turkish version of the Test of Early Language Development (TELD‐3) was used to evaluate language development in both groups.

In the second session, which occurred within the same week, CIAT was administered to participants who met the inclusion criteria for the study. All tests were conducted by an audiologist in a quiet environment. Each participant completed all relevant subtests of the CIAT battery appropriate to their chronological age and auditory perception level. After two weeks, the test was repeated for 72 randomly selected participants to assess test–retest reliability.

#### 2.3.1 Test of Early Language Development

Language development was assessed using the TELD‐3, which includes two subtests: receptive language and expressive language. The test was administered in mixed auditory‐verbal settings and measures language skills in children aged 2–7 years and 11 months (Güven and Topbaş 2014). Due to the lack of other standardized language tests for older children, the final items of the TELD‐3 were also given to children aged 8 and older. In addition, the age‐equivalent scores and standard scores of the subtests were recorded.

#### 2.3.2 Children's Auditory Perception Test

CIAT was developed in Turkish to evaluate the auditory perception skills of children aged 2–15. It consists of 17 tests divided into 6 different categories. The categories are:

1. Category: Phoneme Detection Test

(i) Subtest 1a: Phoneme Detection Test

1. Category: Pattern Identification Test

(i) Subtest 2a: Differentiating Synthetic Syllable Structures Test
(ii) Subtest 2b: Identification Synthetic Syllable Structures Test
(iii) Subtest 2c: Pattern Perception Test (this test consists of both standard and subversions)

1. Category: Speech Identification Test (closed‐set)

(i) Subtest 3a: Word Identification Test (these tests consist of both standard and subversions)
a. Subtest 3a.1. Trisyllable Word Identification Test
b. Subtest 3a.2. Monosyllabic Word Identification Test
(ii) Subtest 3b: Sentence Identification Test
(iii) Subtest 3c: Mrs. Potato Head Test

1. Category: Auditory and Visual Integration Test (it consists of two lists: List A and List B)
2. Category: Modified Open‐Set Speech Identification Test (it consists of two lists: List A and List B)
3. Category: Open‐Set Speech identification and Recognition Test

(i) Subtest 6a: Turkish Sentence Test (this test consists of both standard and subversions, with each version containing six lists: List A to List F)
(ii) Subtest 6b: Listen and Do Test (this test consists of both standard and subversions, with each version containing six lists: List A to List F)

In the test battery, the maximum scores for each test category varied. To calculate the total auditory perception score and to enable accurate comparison between categories, raw scores were normalized to a range of 0–100. This normalization process ensures that variables measured by different tests are in the same range, preventing any single variable from becoming disproportionately influential (Han et al. 2013).

Since the maximum total score that can be obtained from each test differs, participant scores were adjusted to fall within a 0–100 range across all tests. The following formula was applied to all tests for this purpose:

zi=(xi−−min(x))/(max(x)−−min(x))×100

where *zi* is the *i*th normalized value in the data set, *xi* is the *i*th value in the data set, min(*x*) is the smallest value in the data set, and max(*x*) is the largest value in the data set.

### 2.4 Statistical Analysis

The Pearson chi‐square test was used for categorical variables, and Student's *t*‐tests were used for continuous variables to investigate differences in demographic and clinical variables between the control and study groups, as well as differences in auditory perception test scores across demographic groups. Descriptive statistics of clinical and demographic characteristics are presented as frequency, percentage, mean, and standard deviation, depending on the distribution. For comparisons between control and study groups that did not show a normal distribution, the Mann–Whitney *U* test was applied. In tests that fall under the same basic category and share the same minimum and maximum scores, equivalences between categories were checked to represent a large number of test items with fewer tests. Equivalence analyses were performed using RStudio programming. All other statistical tests and graphs were implemented by BM SPSS Statistics (Version 23).

To determine the stability of the results obtained from the auditory perception test over time, the reliability coefficient was calculated using the test–retest method. For this purpose, data were collected from the same group after a two‐week interval, and the consistency between the measurement results was examined using the intraclass correlation coefficient (ICC). Cronbach's alpha reliability analysis was performed to assess the internal consistency of the items in the total score and subcategories of the auditory perception test.

To evaluate known‐group validity, a type of construct validity, the Mann–Whitney *U* test was employed by comparing the auditory perception scores of normal‐hearing children and CI users across age groups. In addition, predictive validity was assessed through multiple linear regression analysis, exploring the relationship between clinical features and auditory perception scores.

## 3 Results

The control group performed statistically higher in the receptive language skill (*p* < 0.001) and expressive language skill (*p* < 0.001).

### 3.1 Equivalence Analysis Results

The equivalences between categories were examined, and the results are presented in Appendix 1.

The equivalence analysis of Lists A and B in the Auditory and Visual Integration Test showed that the equivalence test was not significant, with *t*(358) = −0.0134 and *p* = 0.989. Therefore, the difference between Lists A and B was not significant at the 5% level. Similarly, the equivalence analysis for Lists A and B in the Modified Open‐Set Speech Identification Test was not significant. In the Open‐Set Speech Identification Test category, the Turkish Sentence Test (both subversion Lists A–F and standard version Lists A–F) showed insignificant *p*‐values. Similarly, for the Listen and Do Test, both the subversion Lists A and B and the standard version Lists A and B also had insignificant *p*‐values.

Based on these findings, as the lists showed similar scores, only the first lists from each category of tests were used in the analysis.

### 3.2 Reliability Analysis

Reliability analysis of the total test battery and its subtests, including test–retest reliability and item‐total correlation results, are presented in Table 2. The Cronbach's alpha reliability coefficient for all 17 tests was 0.913. The Cronbach's *α* coefficients in the subcategories ranged from 0.741 to 0.973, indicating that the internal consistency of all categories ranged from acceptable to excellent reliability (*α* = 0.913 > 0.90).

**TABLE 2:** Internal consistency and test–retest reliability of the CIAT.

		Test–retest reliability	Item‐total correlation	Internal consistency
		Intraclass correlation (*p* value)		Cronbach *α* coefficient
Phoneme Detection	PD	—	0.914	—
Pattern Identification	DSSS	—	0.834	0.90
	ISSS	0.514 (< 0.001)	0.854	
	PP	—	0.944	
	PP‐S	—	0.871	
Speech Identification	TWI	0.496 (< 0.001)	0.952	0.965
	MWI	0.747 (< 0.001)	0.951	
	SI	0.585 (< 0.001)	0.952	
	PHT	0.938 (< 0.001)	0.968	
	TSWI‐S	—	0.965	
	SSWI‐S	0.712 (< 0.001)	0.960	
Auditory and Visual Integration	AVI	0.863 (< 0.001)	0.903	—
Modified Open‐Set Speech Identification	SI‐MO	0.228 (0.054)	0.917	—
Open‐Set Speech Identification and Recognition	TST	—	0.900	0.741
	TST‐S	0.962 (< 0.001)	0.923	
	LD	0.914 (< 0.001)	0.907	
	LD‐S	0.874 (< 0.001)	0.907	
	General			0.913

Abbreviations: AVI, auditory visual integration test; DSSS, Differentiating Synthetic Syllable Structure test; ISSS, Identification Synthetic Syllable Structure test; LD‐A, Listen and Do Test standard version; LD‐S, Listen and Do Test subversion; MWI, Monosyllabic Word Identification test standard version; MWI‐S, Monosyllabic Word Identification test subversion; PD, Phoneme Detection test; PHT, Mrs. Potato Head test; PP, Pattern Perception standard version; PP‐S, Pattern Perception test subversion; SI, Sentence Identification test; SI‐MO, Modified Open‐Set Sentence Identification test; TST, Turkish Sentence Test; TST‐S, Turkish Sentence Test standard version; TWI, Trisyllable Word Identification test standard version; TWI‐S, Trisyllable Word Identification test subversion.

### 3.3 Test–Retest Reliability

Test–retest reliability was assessed in 72 randomly selected subjects with a 2‐week interval between tests. This interval was deemed appropriate, as significant changes in speech perception skills are unlikely within such a short period. The reliability values varied between 0.228 and 0.962. For the Phoneme Detection, Differentiating Synthetic Syllable Structure, Pattern Perception, Pattern Perception Subversion, and Trisyllable Word Recognition Subversion tests, the scores obtained in the retest were identical to the original test, resulting in zero variance and an inability to calculate a test value. However, approximately 98% of the observations received the same total score in the Phoneme Detection Test. Similarly, 100% of the observations in the Differentiating Synthetic Syllable Structure Test and 98% in the Pattern Perception Test achieved the same highest score. In the Pattern Perception Subversion and Trisyllable Word Recognition Subversion tests, 98% of the observations matched their previous test scores. Likewise, 96% of the observations received the highest score in the Turkish Sentence Test (subversion). All calculated item‐total correlations for the tests were greater than 0.50, indicating that all 17 tests significantly contributed to the overall scale. Therefore, all items in the scale effectively measure the same construct.

### 3.4 Validity Analysis

#### 3.4.1 Known‐Group Validity

**FIGURE 1:**
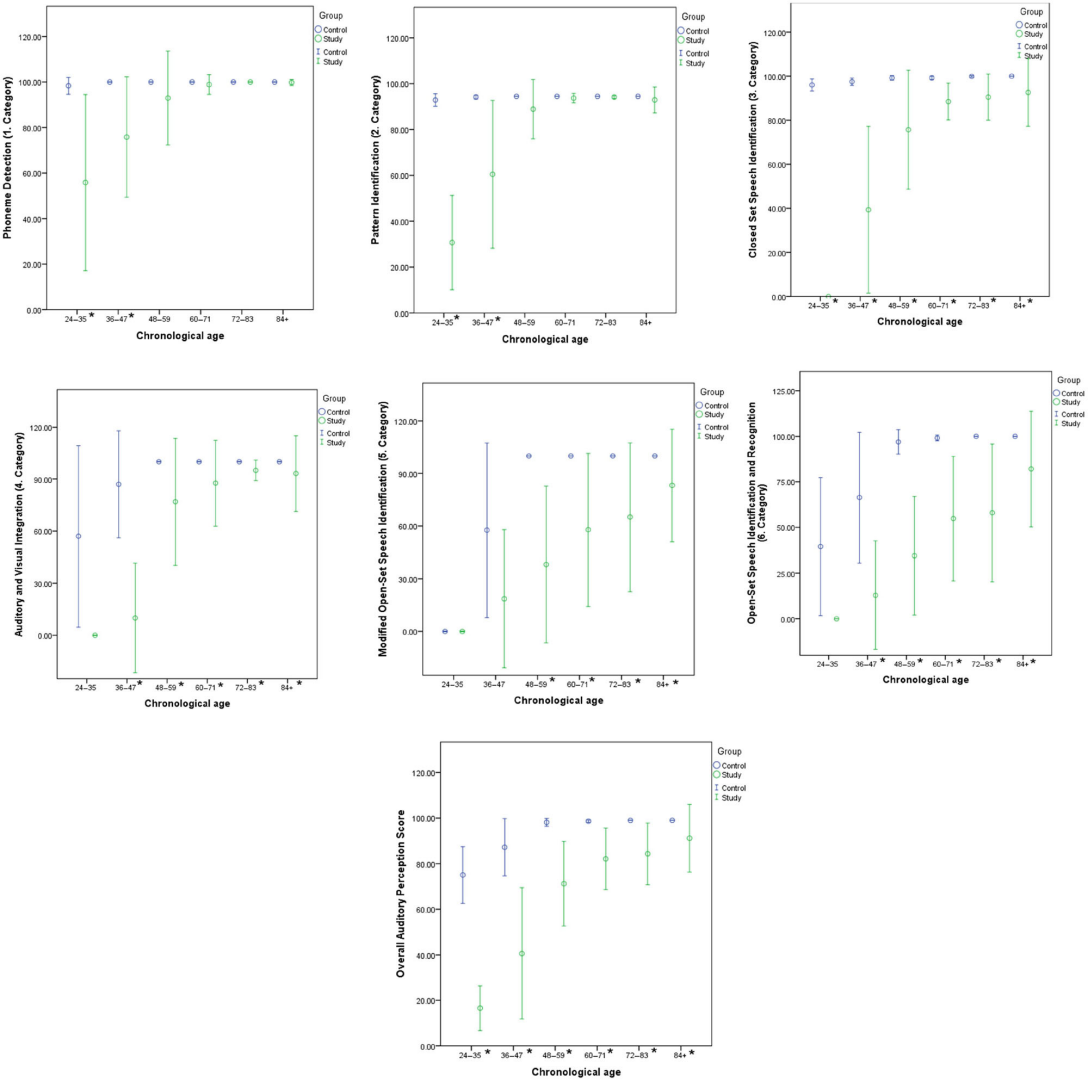
(a–g) Error plots (mean ± standard deviation) of auditory perception test score by chronological age. *p value < 0.05.

To evaluate the known‐group validity, a type of construct validity, the score differences between the control group and the study group were tested across different age groups. The results of this analysis are presented in Figure 1a–g. According to the findings, in the 24–35‐month age group, the total scores of the control group were significantly higher than those of the study group in the first three categories (*p* < 0.05). For the 36–47‐month age group, significant differences were observed between the two groups in all categories except for the fifth category (*p* < 0.05). In older age groups (48 months and above), no significant differences were found between the two groups in the first two categories, while the control group achieved significantly higher mean scores than the study group in the subsequent categories (*p* < 0.05). These differences in mean scores provide evidence of the test's discriminative ability.

### 3.5 Predictive Validity

In predicting auditory perception scores, a multiple linear regression analysis was conducted including factors such as chronological age group, gender, special education period, receptive language equivalent age, expressive language equivalent age, duration of CI, and CI usage status. The results of this analysis are presented in Table 3.

**TABLE 3:** Multiple linear regression analysis of auditory perception score.

						95% confidence interval	
	B	SD	Std.B	*t*‐value	*p*‐value	Lower	Higher
Age	5.02	1.46	0.303	3.42	0.001	2.108	7.940
Duration of CI	8.29	1.58	0.346	5.24	0.000	5.163	11.459
CI usage status	5.68	1.71	0.183	3.47	0.001	2.541	9.341
Age of expressive language	0.29	0.07	0.320	3.85	0.000	0.140	0.439
F statistic	84.93	*p* < 0.001					
*R* (Elliott and Katz, 1980)	0.78						

Abbreviations: B, estimation of coefficient, *R* ^2^, coefficient of determination, SD, standard deviation.

**APPENDIX 1:** Equivalence analysis results.

			$\bar{X}\pm \mathrm{SS}$	Median	Minimum	Maximum	*p*‐value
	Auditory and Visual Integration Test	List A	16.59 ± 7.07	20	0	20	0.989
		List B	16.60 ± 7.07	20	0	20	
	Modified Open‐Set Speech Identification Test	List A	35.42 ± 21.43	49	0	50	0.919
		List B	35.19 ± 21.36	49	0	50	
Open‐Set Speech Identification and Recognition Test	Turkish Sentence Test Standard Version	List A	15.21 ± 8.26	20	0	20	0.918
		List B	15.30 ± 8.28	20	0	20	
		List C	15.33 ± 8.29	20	0	20	
		List D	15.28 ± 8.27	20	0	20	
		List E	15.29 ± 8.28	20	0	20	
		List F	15.28 ± 8.28	20	0	20	
	Turkish Sentence Test Standard Version	List A	28.60 ± 21.26	43	0	46	0.943
		List B	28.76 ± 21.26	44	0	46	
		List C	28.68 ± 21.35	44	0	46	
		List D	28.73 ± 21.37	44	0	46	
		List E	28.70 ± 21.35	44	0	46	
		List F	28.67 ± 21.35	44	0	46	
	Listen and Do Test Subversion	List A	7.62 ± 3.96	10	0	10	0.529
		List B	7.36 ± 3.87	9.50	0	10	
	Listen and Do Test Standard Version	List A	6.79 ± 4.15	9	0	10	0.891
		List B	6.85 ± 4.17	9	0	10	

*p < 0.05.

The model identified that the variables significantly affecting the total auditory perception score were chronological age, duration of CI, CI usage status, and expressive language age (*p* < 0.05). Variables were selected using the stepwise regression analysis method. The significant variables in the model accounted for 78% of the explanatory success for the total auditory perception score (*R* ^2^ = 0.78). This model was found to be statistically significant in explaining the dependent variable (*F* = 84.93, *p* < 0.001). According to the estimated regression coefficients:

- A 1‐year increase in the chronological age led to an average increase of 5.02 points in the auditory perception score.
- A 1‐year increase in the duration of CI use resulted in an average increase of 8.29 points in the total score.
- Each change in CI usage status (from unilateral CI to bilateral CI) was associated with an average increase of 5.68 points in the total score.
- A 1‐month increase in expressive language age led to an increase of 0.29 points in the total score.

## 4 Discussion

In our study, the aim was to validate and ensure the reliability of an updated auditory perception test tailored to the characteristics of the Turkish language, incorporating current cultural needs and lifestyle changes in its content and imagery. This study stands out as the first to revisit and enhance an existing test while thoroughly evaluating its validity and reliability in Turkish‐speaking children.

The literature emphasizes the importance of developing tests tailored to specific languages and cultures for evaluating speech perception skills. El‐Dessouky et al. (2019) developed an Arabic assessment chart of auditory skills for children with CI aged 3–6 years. Likewise, Bhimte and Rangasayee (2018) created a speech perception test in Hindi for children aged 3–7 years with normal hearing. Dawson et al. (2022) adapted the English version of the ESP test for children with normal hearing aged 3–6 years, modifying it to meet the linguistic requirements of the Tamil language.

In the present study, both the wide age range of children evaluated and the inclusion of scores from both CI users and normal‐hearing children make it unique. This design allows for a more comprehensive comparison of auditory perception skills across different populations, providing valuable insights into the performance of children with varying hearing abilities. By incorporating both groups, the study can offer a clearer understanding of how CIs impact auditory perception in comparison to typical hearing development.

The Auditory Visual Integration Test, Modified Open‐ Set Speech Identification Test, and Listen and Do Test, as part of the CIAT battery, include two equivalent subtests each. In the Open‐Set Speech Identification Test category, the Turkish Sentence Test's subversion and standard versions offer six equivalent subtests. Statistical analysis showed no significant differences between Lists A and F across all categories, confirming they are equivalent and interchangeable. The availability of equivalent tests within CIAT is a key strength, as it enables assessment of a child's actual performance using different lists, reducing score inflation due to test familiarity within the same session.

In our study, similar to findings in the literature, it was observed that children using CIs lag behind their normal‐hearing peers in receptive and expressive language skills. This outcome is not unexpected, as the auditory input provided by CIs is significantly limited compared to typical acoustic hearing. It has been well documented that, even after years of experience with CIs, children often lag behind their normal‐hearing peers in spoken language proficiency. This variability in spoken language outcomes following cochlear implantation is widely recognized (Niparko et al. 2010), with children with CIs, on average, showing delayed language development even after more than ten years of implant use (Geers and Sedey 2011).

### 4.1 Examination of Reliability

Reliability is an estimate of the instrument's ability to reproduce consistent results (Koo and Li 2016). Reliability tests were conducted to examine whether the questionnaire items were consistent with each other. Cronbach's alpha values, a measure of internal consistency, were calculated. The Cronbach's alpha for the entire battery was 0.913, indicating excellent internal consistency. For the subtests, Cronbach's alpha ranged from 0.741 to 0.973, showing that all tests have good to excellent internal consistency. Test–retest reliability was assessed with a 2‐week interval between tests. It is assumed that any changes in participants' performance during this period could be attributed to variations in their auditory perception abilities or other external factors that might influence the results (DeVon et al. 2007). The reliability coefficients ranged from 0.228 to 0.962. Lower values were due to very low variance in some tests. Specifically, for Phoneme Detection, Pattern Identification, and Trisyllable Word Identification Subversion tests, valid correlation values could not be calculated because the scores from the retests were the same and the variance of the retest values was zero. However, the fact that 96%–100% of participants obtained the same scores in these tests supports the consistency of the tests. Item‐total correlations were all above 0.50, confirming that each test significantly contributes to the overall scale. For an item to be considered acceptable, its item‐total correlation coefficient should not be negative, and the acceptable item‐total correlation value should be greater than 0.30 (Cláudia de Souza, Neusa Maria, and Guirardello 2017). These results show that CIAT is a stable and reliable tool for measuring auditory perception. Overall, the test battery demonstrates strong reliability in both internal consistency and temporal stability, making it suitable for clinical and research use.

### 4.2 Examination of Validity

Validity is the degree to which a measurement tool can accurately measure what it is intended to measure (Alpar 2016). In this context, known‐group validity and predictive validity were evaluated in our study.

The known‐group validity analysis demonstrated that the CIAT is effective in distinguishing between children who use CI and those with normal hearing. In this context, the primary purpose of using the known‐group validity analysis is not to predict hearing loss or measure diagnostic accuracy, but rather to emphasize how useful the CIAT is as a tool for differentiating known groups based on auditory perception abilities. Moreover, no study in the literature comprehensively evaluates auditory perception skills across a wide age range for both normal‐hearing children and children using CI, according to age groups.

In the Phoneme Detection Test and the Pattern Identification Test, no statistically significant differences were observed in children older than 48 months. Although children with CI have caught up to their normal‐hearing peers in the initial stages of auditory perception skills, they lag behind in word identification and recognition stages. In the Auditory Visual Integration Test and the Open‐Set Speech Identification and Recognition Test, no statistically significant differences were found between CI users and normal‐hearing children in the 24–35‐month group. Similarly, in the Modified Open‐Set Speech Identification Test, no significant differences were observed between the 24–35‐month and 36–47‐month groups. These findings are thought to be due to the difficulty level of these tests, considering the language development levels of children in this age group.

It is well known that several factors influence the success of CIs. Some of these factors include the onset of hearing loss, residual hearing, duration of hearing loss, age at implantation, duration of CI use, preoperative auditory performance, as well as educational and environmental factors (Demir et al. 2019; Geers 2006). The measurement of speech perception provides direct evidence of the assistance offered to the individual by the CI (Dowell et al. 2002). In our study, the predictive success of chronological age, duration of CI use, CI usage status, and expressive language age in explaining auditory perception scores was found to be 78%. The most influential of these variables is the duration of CI usage. This analysis demonstrates the strong predictive validity of these factors for auditory perception scores.

The degree of hearing loss has been shown to negatively correlate with auditory perception abilities, and the type of hearing technology used significantly influences these abilities (Blamey et al. 2001). To ensure group homogeneity, all participants in the study group were selected from CI users. The main limitation of this study is that the study group consisted solely of children using CI. This decision was made to focus specifically on the auditory perception abilities of CI users, as the test was designed primarily to address the unique needs of this population. However, this limitation means that the effect of the degree of hearing loss on auditory perception performance, as well as potential differences between CI users and hearing aid users, could not be effectively evaluated. Future studies should include children using hearing aids and those with varying degrees of hearing loss to provide a more comprehensive understanding of auditory perception across different groups. In addition, similar research should be conducted with adult populations to further explore the impact of varying degrees of hearing loss and expand the applicability of these findings.

A more comprehensive evaluation of hearing capacity should include a Speech‐In‐Competition (Noise/Babble) Test (Iliadou et al. 2024). One limitation of this study is the absence of real‐life speech perception assessments, such as speech‐in‐babble perception, dichotic digits, and temporal processing evaluation. Future studies should incorporate these measures to better address real‐life auditory challenges.

The use of our test in measuring the effectiveness of auditory interventions and long‐term follow‐up studies will provide significant contributions to determining the prognostic value of the test. In conclusion, this study demonstrates that the CIAT is a reliable and valid tool for assessing auditory perception in children, establishing a foundation for applying its clinical and educational use. Future research should expand the test's application to diverse populations and adapt it to various languages.

## 5 Conclusion

The CIAT has been established as a valid and reliable test battery in the literature, designed to assess auditory perception skills such as detection, pattern perception, identification, and recognition at varying difficulty levels across a wide age range, from 2 to 15 years.

## Author Contributions

**Ozlem Icoz**: conceptualization, methodology, data curation, investigation, validation, writing–original draft, resources, funding acquisition. **Selen Yilmaz Isikhan**: formal analysis, funding acquisition, visualization. **Esra Yucel**: writing–review and editing, project administration, supervision, resources, funding acquisition.

## Ethics Statement

This study was approved by the ethical committee of Hacettepe University (GO 17/991 2018/03‐20).

## Consent

Before beginning the study, all parents and children were given verbal and written information about the purpose of the study, and their written information consent was obtained.

## Conflicts of Interest

The authors declare no conflicts of interest.

### Peer Review

The peer review history for this article is available at https://publons.com/publon/10.1002/brb3.70301.

## Data Availability

The data supporting the results reported in the manuscript are kept by the first author. Where appropriate, data analyzed or generated during the study may be shared.
